# Metallographic and Mechanical Research of the O–Ti_2_AlNb Alloy

**DOI:** 10.3390/ma13133006

**Published:** 2020-07-06

**Authors:** Joanna Małecka, Dariusz Rozumek

**Affiliations:** Faculty of Mechanical Engineering, Opole University of Technology, 45-271 Opole, Poland; d.rozumek@po.edu.pl

**Keywords:** microstructure, fatigue, propagation, notches

## Abstract

This paper provides the test results of the fatigue crack growth in notched specimens under tension. The tests were performed on plane specimens with external blunt two-sided notches at room temperature. The tested material was O–Ti_2_AlNb titanium alloy. The tests were carried out at constant load value and constant stress ratio R = 0. The tests were done at the fatigue stand Instron under the loading change frequency of 25 Hz. The results of mechanical and structural tests of the examined alloy were presented. Scanning electron microscopy (SEM) was used in metallography, which allowed for the initial identification of intermetallic phases. The development of fatigue cracks in the tested alloy indicates that there is a tendency for brittle fracturing, which ran along the grain boundary.

## 1. Introduction

Unique physical and mechanical properties of titanium and its alloys, relative to other construction materials, are the reasons for its wide used in industrial fabrication. Still, the development of technology tightens up the engineering materials requirements, at the same time ensuring competitiveness at the global level. The development of technology places ever increasing demands on engineering materials. Materials with low density, relatively good resistance to oxidation, but at the same time, maintaining good mechanical properties are becoming more and more popular.

Titanium alloys belong to such a group of materials, which are characterized by high strength and good corrosion resistance, however, due to poor resistance to oxidation, their use is limited. Alloys with based on intermetallic phases [1,2,3] as have become an alternative traditional titanium alloys. These alloys include: Ti_3_Al (α_2_), TiAl (γ), TiAl_2_ and TiAl_3_, which partly overcame the abovementioned limitation. These materials were to replace titanium alloys and nickel-based alloys used in the construction of jet engines [2,3,4]. However, only TiAl (γ) and Ti_3_Al (α_2_) phase based alloys found practical application.

Titanium alloys, which most recently have joined the family of the intermetallic alloys are alloys, based on the intermetallic compound Ti_2_AlNb(O) [5,6] which appeared at the beginning of 1990 as a result of extensive research on alloys Ti_3_Al(α_2_). They are characterized by a higher density but increased ductility at the same time. Nevertheless, it should be noted that these materials have not found practical use yet and are still at the research stage. It seems, however, that due to their relatively favourable properties, they may become an interesting alternative to the currently used materials.

**Figure 1:**
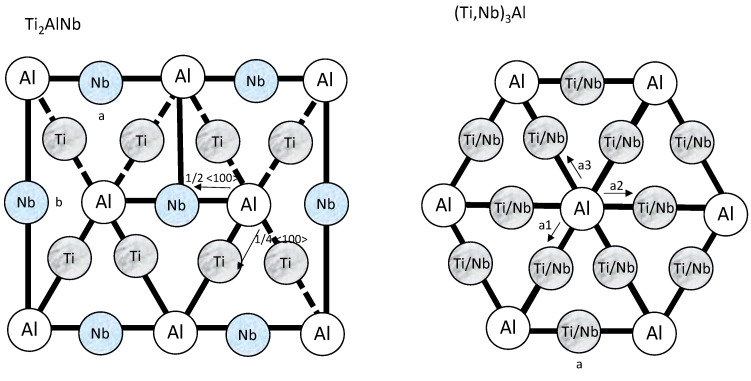
Arrangement of Nb and Ti atoms in network cells (diagrams were elaborated based on Reference [8]).

In the early 1990s, a new alloy was discovered—Ti_2_AlNb. This alloy is often referred to as O–Ti_2_AlNb. The first letter “O” means a type of structure—orthorhombic. The differences consist in the arrangement of Nb atoms in relation to Ti atoms in the crystal lattice, as shown in Figure 1 [5,7].

Replacement of the α_2_-Ti_3_Al phase with the Ti_2_AlNb phase in TiAl alloys increased their density. It was, however, compensated for by improving other properties such as increasing plasticity at room temperature (~3%); low coefficient of thermal expansion ~9.5 × 10^−6^ (700 °C); good compatibility with SiC, enabling the formation of composites (SiC fibers + TiAl-Nb alloy). These materials are still in the research stage and are not commercially produced until now (2020).

The issues that bring together scientists’ efforts primarily concern increasing their resistance to high temperature oxidation, increasing ductility and improving their technological properties [9,10,11,12,13].

Some of the works describe the mechanical and fatigue properties of titanium alloys [14,15,16,17]. In [15], the model to predict the fatigue crack growth rate in Ti–6Al–4V is shown. The equation assumes that the fatigue crack growth rate is directly proportional to the change in displacement of the crack tip opening during fatigue loading between the minimum and maximum stress intensity factor. The test results, obtained for fatigue crack in titanium alloy, and subjected to bending, is presented in [16]. The tested specimens were made of the oxygenated Ti–6Al–4V and subjected to various variants of heat treatment. From the obtained results of the obtained result fatigue and structural tests, it appears that crack propagation and fatigue lives of the considered alloy are different depending on a structure obtained as a result of a given heat treatment. The results of fatigue tests for Ti–6Al–4V alloy are presented in paper [18]. The authors carried out tests both, at room temperature and for specimens cooled with liquid nitrogen. The results obtained determined the value of total strain, for which a change in material properties of the tested material is observed.

Relatively, some of the works describe the protective properties of scale that forms in the course of oxidation titanium alloys, based on intermetallic phases at high temperatures, because in most cases, good protection of the material is desired for a long time [19,20]. In the past few years, research has focused on TiAl alloys with a high Nb content. It was shown in [21], that Nb can improve oxidation resistance. The alloying of Nb, increases the thermodynamic activity of Al relative to that of Ti, which favours the formation of a stable alumina scale. Moreover, in the high temperature oxidation process, the Nb ions replace Ti ions, leading to a decrease in oxygen vacancies and slowing diffusion of oxygen.

There are a number of works concerning oxidation behaviour, but little work has been done on the mechanical properties of titanium alloys, based on intermetallic phases. For this reason, this paper presents metallographic and mechanical tests of the O–Ti_2_AlNb alloy. Static and cyclic tests were performed at tension, and then the cracks growth was analysed.

## 2. Material and Testing Procedures

### 2.1. Material and Specimen

The tests were performed on O–Ti_2_AlNb based Ti–25Al–12.5Nb (at. %) alloy with a content of β-stabilising elements. The chemical composition of the analysed material are shown in Table 1.

In the O–Ti_2_AlNb alloy, niobium is the most effective alloying element improving plasticity in room temperature. A characteristic alloy of this generation is the super-alpha 2TM alloy with the composition Ti–25Al–10Nb–3V–1Mo [22].

The microstructure of O–Ti_2_AlNb alloy is depending on heat treatments and processing methods [23]. According to the microstructure classification of orthorhombic alloys, can distinguish: equiaxed microstructure, bimodal microstructure, lamellar microstructure, and lamellar microstructure with coarse secondary O laths and thick grain-boundary α_2_ phase.

**Figure 2:**
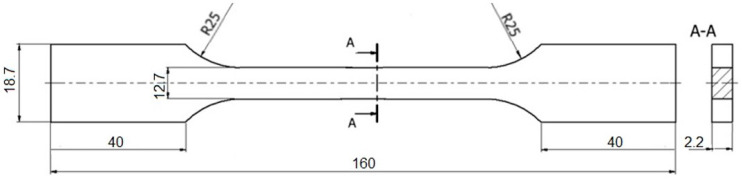
Shape and dimensions of specimens, dimensions in mm.

The static and fatigue tests were performed for specimens which shape and dimensions of are shown in the Figure 2. Specimens were cut using Wire Electrical Discharge Machining (WEDM) method. The plane specimens had stress concentrators in the form of external blunt two-sided notches, of 3.0 mm in depth, the radius of the notch root rounding R = 25 mm, on which the next crack growth was considered in the structural aspect of the material.

The specimens (Figure 2) were cut from the sheet with a thickness of 4 mm according to the rolling direction. The specimen surface have been obtained by milling and grinding with abrasive paper. The notches were made with the milling cutter, and the specimen surface was grinded.

The fatigue life of O–Ti_2_AlNb alloy is significantly affected by the surface roughness. Thus, the arithmetic mean roughness Ra and the maximum height of the roughness Rz were measured using a TOPO-01P profilometer (Digital Surf Companies, Besançon, France) with a diamond stylus radius of 2 µm. Surface profiles/topographies were recorded and 3D roughness parameters were estimated on the scanned areas of 1.0 mm × 1.0 mm and the values of the Ra and Rz were measured. To obtain the mean value and the standard deviation, the surface was measured three times at each case.

Microstructural analyses and the cross-section of the tested specimens after the test was finished, were characterized by scanning electron microscopy, using a JEOL JSM-840A microscope (JEOL Companies, Tokyo, Japan) microscope equipped with energy dispersive X-ray spectroscopy (EDS) analyser (JEOL Companies, Tokyo, Japan). Secondary electrons (SE, JEOL Companies, Tokyo, Japan) were used in the analyses. The specimens were etched with reagent to provide structure investigation, as follows:

- 30 mL C_3_H_6_O_3_ (Struers Companies, Cracov, Poland)
- 15 mL HNO_3_ (Struers Companies, Cracov, Poland)
- 5 mL HF (Struers Companies, Cracov, Poland)

The analysis of the phase composition of the tested alloy was carried out with the use of X’Pert PRO X-ray machine (Panalytical Companies, Malvern, UK) with an X’Celerator strip detector (Panalytical Companies, Malvern, UK) (Bragg-Brentano system, Panalytical Companies, Malvern, UK).

### 2.2. Static and Fatigue Testing

The experimental tests were carried out at the INSTRON 100 kN fatigue machine (Instron Companies, Norwood, MA, USA) with a hydraulic drive [24]. The INSTRON machine is used for testing specimens made of structure materials under uniaxial loads. This device allows you to perform tests at maximum static load and cyclically variable loads up to 100 kN. The tests were performed with a controlled force for a constant load amplitude tension F_a_ = 1.6 kN which corresponded to the nominal stress value in the specimen cross-section of 72.7 MPa. The mean load value was F_m_ = 1.6 kN (P_max_ = 3.2 kN). Synchronous sinusoidally variable loadings were applied. The tests were carried out for the constant stress ratio R = σ_min_/σ_max_ = 0 and a load frequency of 25 Hz. The course of changes taking place in the specimen was observed and recorded on a computer. The number of loading cycles until specimen failure was also recorded. A static tensile test was performed and the obtained result showed a yield strength of 106 MPa and tensile strength of 125 MPa while its microhardness is 446 HV.

## 3. Results and Discussion

### 3.1. Metallographic Analysis

**Figure 3:**
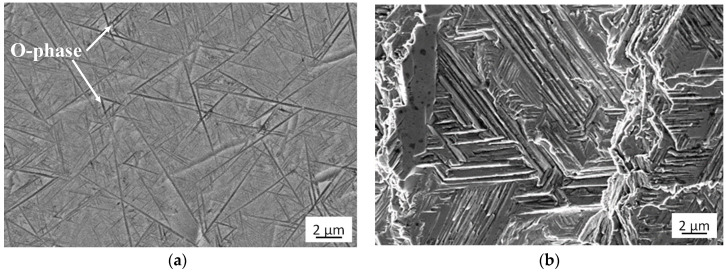
Structure (**a**) and fracture (**b**) of Ti–25Al–12.5Nb–6.01Mo–0.48V alloy.

The structure and fracture of the tests alloy is presented in Figure 3 and such a structure type can be specified as lamellar. It is a typical microstructure generated as a result of beta heat treatment of the material.

**Figure 4:**
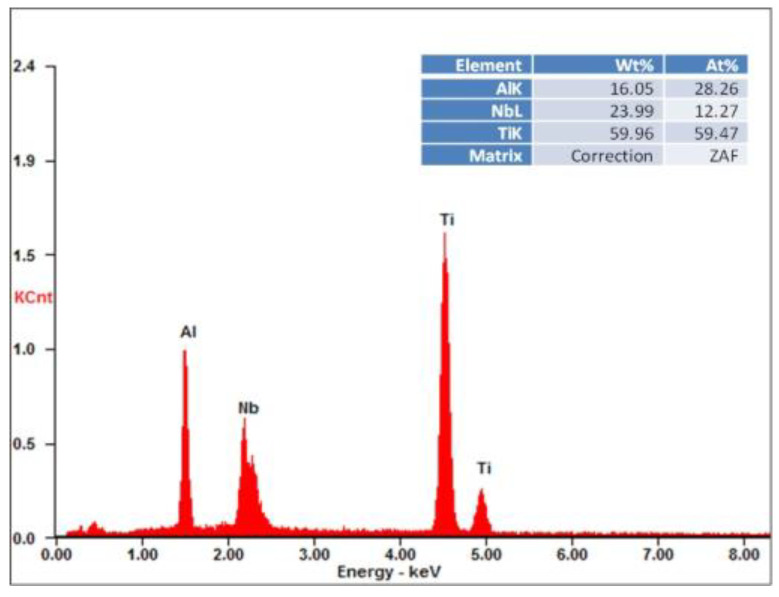
EDS analysis results of tested alloy.

**Figure 5:**
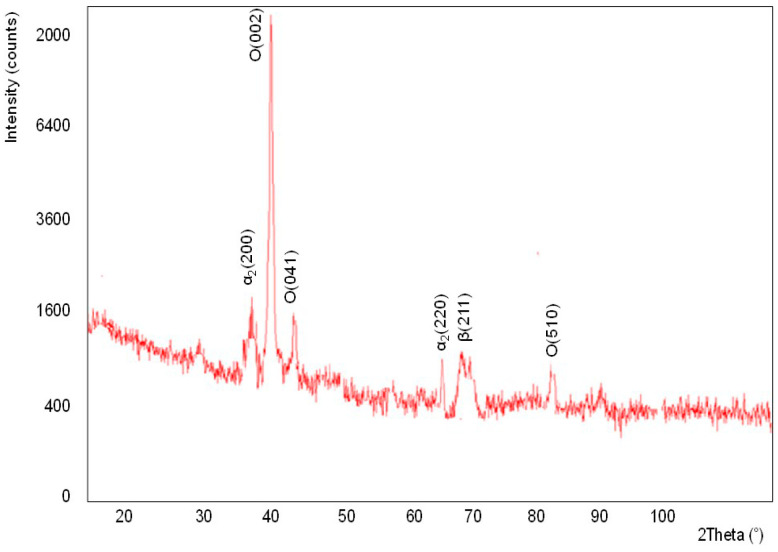
XRD analysis result of tested alloy.

The EDS analysis of the chemical composition of the surface of tested alloy (Figure 4) proved that the composed mainly of Al, Ti and Nb. The applied method of X-ray qualitative phase analysis carried out in Bragg-Brentano geometry confirms the presence of the respective phases in the material (Figure 5): α_2_, β and O–Ti_2_AlNb.

**Figure 6:**
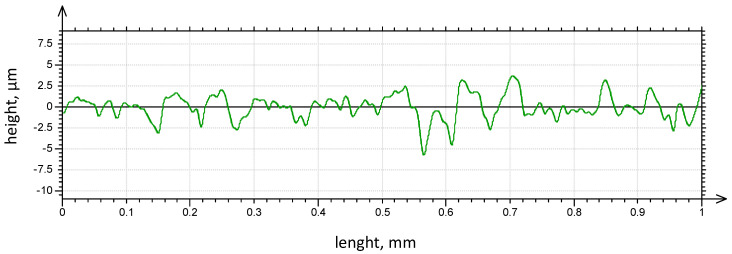
The surface profiles of the specimen.

**Figure 7:**
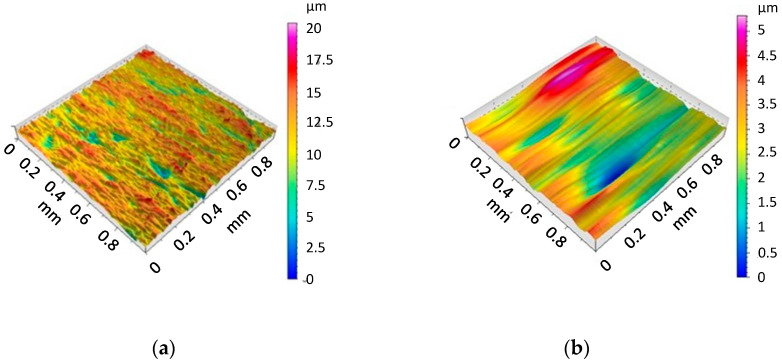
The surface roughness (**a**) and waviness (**b**) of the specimen.

The values of the Arithmetical Mean Roughness Ra and Rz measured on the surface of specimens were obtained as follows: Ra = 1.21 µm and Rz = 6.86 µm. The The surface profiles of the specimen is presented in Figure 6 and the surface roughness and the surface waviness in Figure 7.

By reducing roughness on the surface of the material, we get rid of micro-notches that are the initiators of fatigue cracks [25]. If we change the surface roughness from Ra = 1.25 to Ra = 0.32, we increase the fatigue life of the element up to three times.

### 3.2. Material after Static Testing

The microstructure of materials has a large impact on the rate of fatigue crack growth and has a decisive role in the propagation of cracks [26]. The analysis of surface, static and fatigue fractures of the specimens, carried out by means of SEM microscopy, revealed the location of the source of cracking, and these observations provided more details on the place of formation and mechanism of crack growth, both static and cyclic.

**Figure 8:**
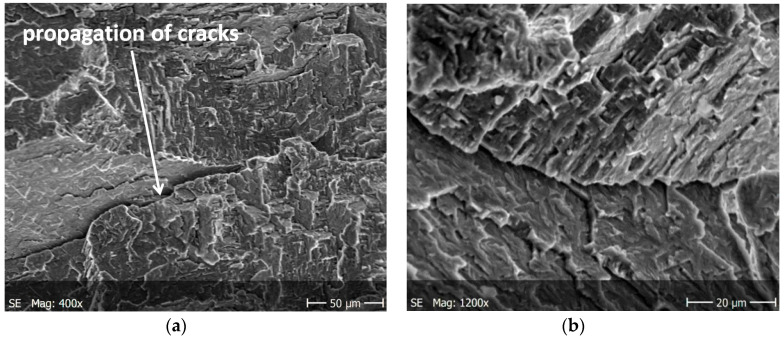
Cracking of O–Ti_2_AlNb alloy in specimen after static tests for (**a**) a larger specimen area, (**b**) enlarged crack.

**Figure 9:**
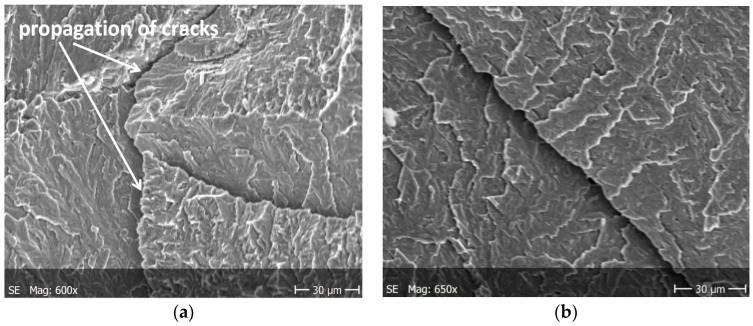
Cracking of O–Ti_2_AlNb alloy in specimen after static tests for (**a**) a larger specimen area, (**b**) enlarged crack.

Brittle cracks dominate on both surfaces of the specimens subjected to static tension. Lamellar layers form during cracking. Figure 8a and Figure 9a show a larger area of the specimen to see how it propagates the crack, while these are enlarged areas to analyze the crack.

**Figure 10:**
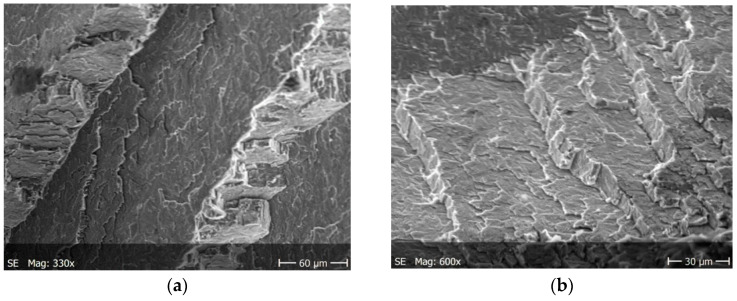
Fractures in the tested material after static tests for (**a**) a larger specimen area, (**b**) enlarged crack.

Figure 10 shows static fractures in the tested material. Lateral cracks are characterized by anisotropy with respect to the axis of the specimen and that they form characteristic stacking faults. At the fracture of Figure 10, you can also see faults, lamellar cracks.

### 3.3. Development of Fatigue Cracks

**Figure 11:**
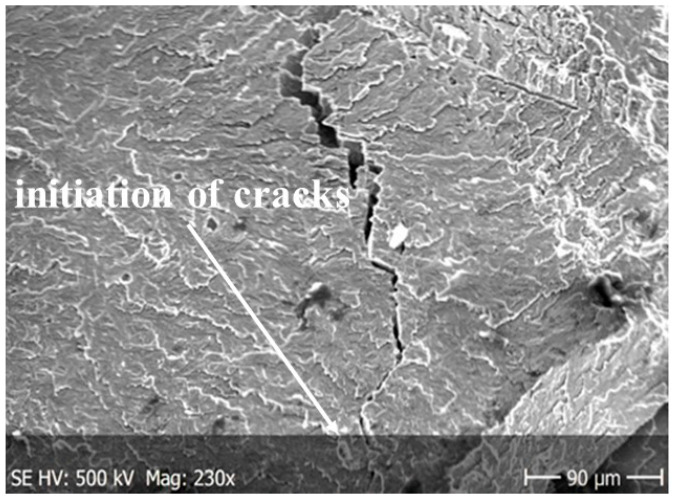
Initiation of cracks in cyclic tests.

**Figure 12:**
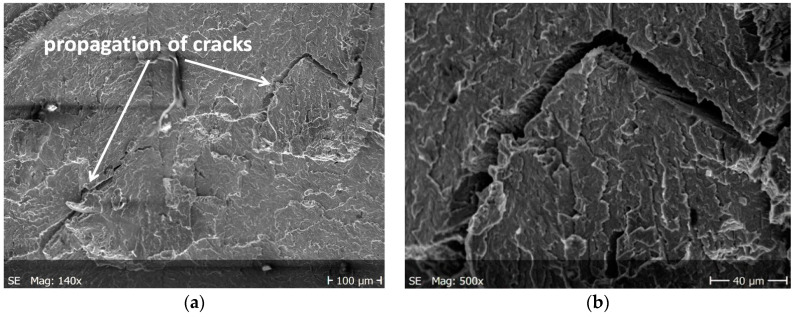
Cracking of O–Ti_2_AlNb alloy in specimen after cyclic tests for; (**a**) a larger specimen area, (**b**) enlarged crack.

**Figure 13:**
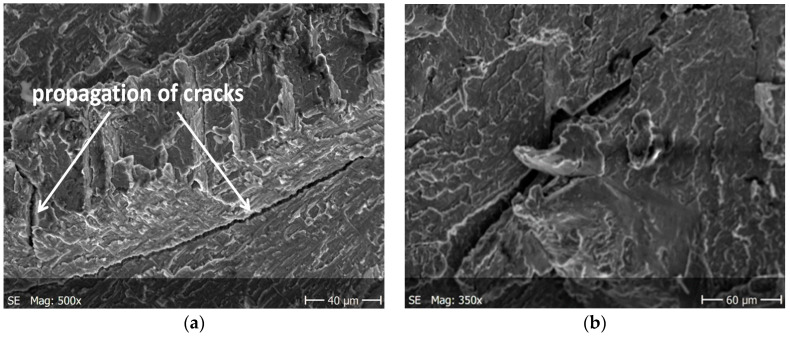
Cracking of O–Ti_2_AlNb alloy in specimen after cyclic tests for (**a**) a larger specimen area, (**b**) enlarged crack.

The specimens shown in Figure 2 were used for the fatigue crack growth tests. For the load P_a_ = 1.6 kN tests were performed under cyclic tension, obtaining the number of cycles to failure N_f_ = 124,130 and 163,050, respectively. The initiation of cracks is shown in Figure 11, and the crack formation during the cyclic tests of the O–Ti_2_AlNb alloy is shown in Figure 12 and Figure 13.

The main cracks initiate and developed in net cross-sections of specimens. The propagation of short secondary cracks from the main crack on the surface of the sample fracture is characteristic. In addition, small faults were observed in front of the front of the main crack. This causes the cracks to form a zigzag pattern (Figure 12a). The main crack growth may be formed by initially formed micro-cracks propagating in the directions of tangential stresses and then their joining to form the long, main crack. In addition, the characteristic feature of secondary cracks is their multi-directionality with respect to the axis of the specimen and the creation of characteristic stacking faults.

In both specimens, irregular cracks were observed at cyclic loads. The cracks propagating mainly along the grain boundaries (Figure 12b and Figure 13b). Figure 13a shows a fairly long linear crack, which may indicate the rate of the developing crack. The reasons for this behaviour is to be found in brittle of the tested material, in which propagating cracks tend to a stress relaxation.

**Figure 14:**
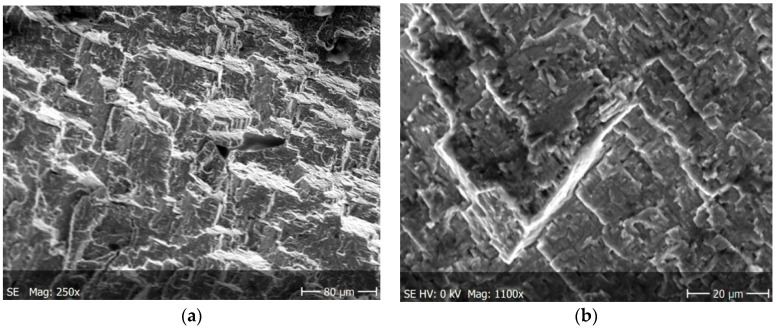
Fractures in the tested material after fatigue tests for; (**a**) a larger specimen area, (**b**) enlarged crack.

The fractures shown in Figure 14 are characterized by clear granularity regardless of the applied test methodology. However, the cleavage areas that appear in the fractures may indicate a tendency to brittle cracking of the studied alloy. It is observed from the aforementioned results that the hardness of brittle intermetallic phases like Ti_2_Al was the highest. Furthermore, the analysis of the fracture surfaces and the tendency of cracks propagation also showed that the cracks were most inclined to be generated in the Ti_2_Al phase when the specimen was subjected to a cyclic tensile force.

## 4. Summary and Conclusions

The process of fatigue developing in structural materials under the influence of loads is still an important problem of modern technology. One of the most important external factors influencing the resistance of materials to cracking is the way they are loaded, and the pattern of the cracking process depends on the overall structural phenomena occurring during the deformation of the material. Moreover, de-cohesion of the structural elements is significantly influenced by the type of material used and operating conditions. The simplest case of de-cohesion is the breakage of the specimen during a static tensile test. However, it should be noted that the large variety of external factors and structural phenomena occurring in the material causes that the history of each decohesion process should be considered separately.

In the presented paper the fractographic analysis indicate a tendency to brittle cracking, which results in the formation of transcrystalline cleavage fractures regardless of the applied research methodology. The surface of the fracture is oriented perpendicularly to the direction of the applied load, without noticeable traces of plastic strain of the material in the macroscale.

The nucleation points of the cracks are the grain and phase boundaries, and the created microcracks spread through the grains simultaneously in several parallel and closely located crystallographic planes, with subsequent formation between them of stacking faults (steps) visible on the surface of the fracture (Figure 10). The surface of the observed fracture is glossy because the planes of the cleavge fracture of material are very smooth. Such areas are visible as separate flat areas of cleavage cracking that are differently oriented, in relation to the fracture plane (Figure 12). On the surface of these flat fracture zones, it is possible to identify the course of secondary cracking with the formation of faults and bridges between adjacent cracks, developing in parallel planes of cleavage (Figure 8 and Figure 9). The faults between the cracks in the parallel planes of cleavage inside one grain or other component of the microstructure form a characteristic fracture pattern (Figure 14). Small, elementary faults merge into larger ones, in the direction of the crack propagation and are the source of the main crack. The stacking faults of the structure may be formed by cleavage cracking along secondary planes, twin boundaries in layers between cleavage cracks. When a cleavage crack occurs at one point, it spreads in all directions in the grain and creates a fan-shaped pattern (Figure 8b). In the observed specimens with fatigue cracks, we can also see secondary cracks, running perpendicularly to the crack propagation direction (Figure 13a).

Comparing the microhardness of a typical Ti–6Al–4V alloy of 344 HV [16], it was about 23% lower than the microhardness of the O–Ti_2_AlNb alloy. The greater hardness of the O–Ti_2_AlNb alloy makes it more brittle than the Ti–6Al–4V alloy [27] and this affects fatigue life. The fatigue life of the Ti–6Al–4V alloy is about 10 times greater than the durability of the O–Ti_2_AlNb alloy. The higher tensile strength of the Ti–6Al–4V [27] alloy by about 87% compared to the O–Ti_2_AlNb alloy coincides with higher fatigue life. In the Ti–6Al–4V alloy, aluminum is only dissolved in the α phase and strongly strengthens it, which means that the degree of strengthening of the α phase in this case is greater than in the O–Ti_2_AlNb alloy. The difference in the α phase plasticity in the alloys tested and the differences in their phase composition result in a different mechanism of strain and cracking along the entire length of the specimens. The β phase has a much higher plasticity compared to the α phase. In Ti–6Al–4V alloys we deal with a more complex development of cracking than in O–Ti_2_AlNb alloys. Numerous secondary (short) cracks branch off from the main crack in Ti–6Al–4V alloys, which prolong the cracking process and material life.

Based on the results of alloy testing and analysis, we can draw the following conclusions:

1. In the material, mainly trans-crystalline cracks were observed but there are also inter-crystalline cracks along the grain boundaries.
2. The development of fatigue cracks in the tested alloy indicates the tendency for brittle fracture and ran along the grain boundary.
3. The observed fractures were lamellar regardless of the methodology of the research.

## Figures and Tables

**Table 1:** Chemical composition of O–Ti_2_AlNb titanium alloy.

Material	% at				
	Al	Nb	Mo	V	Ti
Ti_2_AlNb(O)	25	12.5	6.01	0.48	balance
